# A replication-incompetent adenoviral vector encoding for HSV-2 gD2 is immunogenic and protective against HSV-2 intravaginal challenge in mice

**DOI:** 10.1371/journal.pone.0310250

**Published:** 2024-12-31

**Authors:** Elisa Rossetti, Marija Vujadinovic, Ella van Huizen, Jeroen Tolboom, Hanneke Schuitemaker, Feng Yao, Roland Zahn, Eirikur Saeland

**Affiliations:** 1 Janssen Vaccines & Prevention, Leiden, The Netherlands; 2 Brigham and Women’s Hospital, Boston, MA, United States of America; Rowan University, UNITED STATES OF AMERICA

## Abstract

Herpes Simplex virus (HSV) is the cause of genital herpes and no prophylactic treatment is currently available. Replication-incompetent adenoviral vectors are potent inducers of humoral and cellular immune responses in humans. We have designed an adenoviral vector type 35 (Ad35)-based vaccine encoding the HSV-2 major surface antigen gD2 (Ad35.HSV.gD2). Immunization of mice with Ad35.HSV.gD2 elicited virus neutralizing antibody titers (VNT) and cellular responses against HSV-2 and HSV-1. While immunity was lower than for CJ2-gD2, both vaccines showed 100% survival against intravaginal challenge with HSV-2 G strain and a strong inverse correlation was observed between HSV-2 infection (as measured by viral shedding) and VNT. A combination of Ad35.HSV.gD2 with Ad35 encoding for gB2 (Ad35.HSV.gB2) resulted in increased VNT and lower infection, compared with Ad35.HSV.gD2 alone. Transfer of immune serum into naïve BALB/c mice before intravaginal challenge confirmed the role of antibodies in the protection of mice against infection although other immune factors may play a role as well.

## Introduction

Herpes simplex virus type 2 (HSV-2) is an important cause of genital herpes and estimates indicate that half a billion people between 15 and 49 years of age are living with a HSV-2 infection worldwide with evidence of increasing prevalence [1]. In the developed world, prevalence of genital herpes caused by HSV type 1 (HSV-1) has been on the rise [2].

HSV-2 infection causes a lifelong disease due to the establishment of latency in the dorsal root ganglia (DRG) with episodes of viral reactivation. Transmission occurs through recurrent episodes of viral shedding from infected mucosae that can occur asymptomatically [3]. Clinical symptoms of a primary infection can be genital blisters or ulcers and symptoms associated with viral reactivation are similar, however usually less severe. Infants born from HSV-2 infected mothers are at risk to become infected during birth (or shortly after) and to develop neonatal herpes, that is a rare but devastating condition. The overall global rate of neonatal HSV is estimated to be 10 per 100,000 live births, with a best estimate of 14,000 cases annually (based on seroprevalence, birth rates and infections in pregnancy) [4]. It has also been demonstrated that the risk to acquire and/or transmit human immunodeficiency virus (HIV)-1 infection is 3-fold higher with an HSV-2 co-infection [5]. Available antiviral drugs may be effective during primary infection in reducing the duration of symptoms, viral shedding, and time to healing, but they fail to prevent severity and frequency of recurrences [6].

There are no approved vaccines currently available for prevention of HSV infection. Several vaccine candidates are in pre-clinical development, for prophylactic and therapeutic purposes. Subunit vaccines based on the surface glycoproteins of HSV have shown promising immunogenicity and efficacy in animal models [7–9] but have failed in clinical efficacy trials [10, 11]. In the Herpevac clinical trial, an AS04 adjuvanted subunit HSV-2 gD2 vaccine showed only 58% vaccine efficacy against genital HSV-1 disease in seronegative women while no efficacy was observed against HSV-2 disease (20% overall efficacy against genital herpes disease) [10]. Protection against HSV-1 correlated with the level of gD2 specific antibodies. Cellular immune responses were mainly composed of CD4+ T cells, whereas CD8+ T cell responses were largely absent [12]. It was concluded from these trials that the elicited HSV-specific antibodies were not sufficient for protection and that cellular responses are likely needed for effective viral clearance and control of viral reactivation from the DRG [12–14]. Furthermore, the vaccine did not elicit antibodies capable of mediating antibody-dependent cellular cytotoxicity (ADCC), which have been suggested to play an important role in HSV-2 clearance [15].

Live attenuated vaccines are known for their potential to induce strong and durable humoral and cellular immune responses. However, high immunogenicity of live attenuated vaccines is generally associated with certain safety risks, not observed for subunit vaccines or other vaccine modalities. A major challenge therefore is to develop a strongly and durably efficacious vaccine with an acceptable reactogenicity profile. Another safety concern of live attenuated HSV vaccines is the risk of reversion to a replication-competent phenotype or to recombination with wild type virus during infection [16]. Viral vectors engineered from adenoviruses (Ad) exhibit many additional beneficial properties as vaccines and gene delivery vehicles, such as the ability to support potent antigen-specific immune responses and ease of manufacturing [17, 18]. Adenovirus types with low seroprevalence, such as human adenovirus type 26 (HAdV26) and adenovirus type 35 (HAdV35), have been shown to induce effective humoral and cellular immune responses in humans [19–22].

Here we describe immunogenicity and protective efficacy of a replication-incompetent Ad35-based HSV-2 vaccine encoding gD2 (Ad35.HSV.gD2) and gB2 (Ad35.HSV.gB2) in mice, using a live-attenuated vaccine candidate as a comparator.

## Materials and methods

### Ethics statement

All mouse experiments were conducted in accordance with the Dutch Animal Experimentation Act and the Guidelines on the Protection of Experimental Analysis by the Council of the European Committee after approval by the Centrale Commissie Dierproeven and the Dier Experimenten Commissie.

### Virus and vaccines

Wild-type (WT) Herpes Simplex Virus-2 (HSV-2) G strain was propagated on Vero cells, cell lysates of the virus were generated, and plaque assayed to assess the final viral concentration of 9,38x10^7^ plaque forming unit (pfu)/ml. The virus was used for in vitro (plaque assay, viral neutralization assay) and in vivo experiments (HSV-2 intravaginal challenge). Wild-type HSV-1 KOS virus (1.92x10^12^ pfu/ml) was kindly provided by Søren Riis Paludan (Department of Biomedicine, Aarhus University, Denmark).

Ad35.HSV.gD2 and Ad35.HSV.gB2 are replication-incompetent, E1/E3-deleted recombinant adenoviral vector vaccines generated at Janssen based on the AdVac^®^ technology [22]. Briefly, the Ad35.HSV.gD2 and Ad35.HSV.gB2 were generated by inserting an expression cassette containing the codon optimized gD2 gene and gB2 gene (HSV-2 G strain), respectively, under the human Cytomegalovirus (hCMV.TetO) promotor and the SV-40 (simian virus-40) polyadenylation sequence in the E1 region of the Ad35 genome. The E3 region of the vectors was deleted and the E4orf6 region of Ad35 was replaced by the Ad5 E4orf6 to allow high scale production of the vectors in the complementing PER.C6® TetR cell line. Finally, the vectors were purified by standard two-step CsCl_2_ gradient and dialyzed in formulation buffer. The Ad35 vector concentration expressed as viral particles(vp)/ ml was determined by optical density (OD) with a final concentration of 1.42x10^12^ vp/ml and 1.58x10^12^ for the gD2 and gB2 vectors, respectively.

CJ2-gD2 is an HSV-2 strain 186-derived ICP0-null mutant-based non-replicating dominant-negative recombinant virus encoding the gD2 gene driven by the TetO-bearing HSV-2 ICP4 promoter and the UL9-C535C gene under the control of the truncated form of hCMVTO [23]. CJ2-gD2 was amplified on V0R-124 cells, a Vero-based complementing cell line stably expressing ICP0 and TetR. Cell lysates of the virus were generated by harvesting infected cells and releasing the virus by freeze-thawing cycles, followed by centrifugation to remove large cellular debris, snap-freezing in liquid nitrogen and storing at -80°C until use. Cell lysates were plaque assayed on V0R-124 to determine a viral titer of 7x10^6^ plaque forming units (pfu)/ml.

Vaccines were tested for bioburden (MicroSafe; Millipore, Bedford, MA) and endotoxin (MicroSafe; Millipore) before use for in vivo experiments. Ad35.HSV.gD2, Ad35.HSV.gB2, and CJ2-gD2 showed bioburden of ≤ 1 colony forming units (cfu)/100 μl and endotoxin of <5 EU/ml.

### Animal experiments

Six to eight-weeks-old naïve specific pathogen-free (SPF) female BALB/c mice (Charles River, Germany 5 mice/group) were immunized intramuscularly (i.m.) (50 μl/ hind leg) with one-dose regimen or a two-dose regimen (4 weeks apart) with Ad35.HSV.gD2 (10^8^ to 10^10^ vp/mouse). Also, an additional group of mice received immunization with CJ2-gD2 (7x10^5^ pfu/mouse) at day 0 and 28 and a group of mice received formulation buffer as control. Blood for serum samples were collected by submandibular bleeding before the start of the experiment and before the second immunization timepoint while at the end of the experiment (at 6 weeks) blood was collected by heart puncture under isoflurane anesthesia. Spleens from individual mice were also collected on the sacrifice day to assess cellular immune responses.

In the intravaginal challenge experiments, mice (10 mice/group) were first immunized i.m. (50 μl per hind leg)_with Ad35.HSV.gD2, Ad35.HSV.gB2 or CJ2-gD2. Dose and regimen are specified in the Results section for each experiment. A group of mice receiving formulation buffer was used as a negative control. Five days prior to challenge mice were injected intraperitoneally (i.p.) with 3 mg/mouse of medroxyprogesterone (MPG), (DepoProvera, Pfizer, New York, USA), to synchronize the estrous cycle. Five days later all groups were sedated with Ketamine-Xylazine anesthesia i.p. and challenged intravaginally with 200LD50 (3.2 x10^4^ pfu/mouse) of the WT HSV-2 G strain. Subsequently, mice were observed for clinical signs up to 21 days. Clinical scores in a range from 0 to 4 were as follows: 0: no signs; 1: slight genital erythema and edema; 2: moderate genital inflammation, single or multiple blisters; 3: purulent genital lesions or ulcers; 4: hind leg paralysis, hind leg limping or unresponsiveness. Mice that reached a clinical score of 3 were observed twice a day to monitor eventual rapid progressing of animals into score 4. Animals were sacrificed when they reached score 4. Humane endpoints were considered when animals showed distress or pain (lack of grooming, unresponsiveness, loss of weight) not directly related to the local mucosal symptoms due to the infection. Vaginal mucosae of challenged mice were swabbed with lightly DMEM (Dulbecco’s Modified Eagle Medium, Gibco) soaked calcium alginate swabs (Puritan, Thermo Fisher Scientific) on days 1, 2 and 4 after challenge. Swabs were placed in 1 ml tubes containing DMEM with 10% Fetal Bovine Serum (FBS, Gibco) and 100 units/mL Penicillin/100 μg/mL Streptomycin (Gibco), kept on ice for 1 h, thoroughly vortexed and stored at -150°C. Viral shedding was assessed by standard plaque assay on Vero cells monolayers. At the end of the study, animals were anaesthetized with isoflurane and sacrificed by cervical dislocation.

To generate serum pools for serum transfer, mice (60 mice/group) were first immunized twice in a 4-week regimen i.m. (50 μl/ hind leg) with Ad35.HSV.gD2 (10^10^ vp/mouse) or with CJ2-gD2 (7x10^5^ pfu/mouse). Two weeks after the second immunization animals were sacrificed, and blood was collected via heart puncture under isoflurane anesthesia. gD2-specific IgG titers were measured in individual serum samples by Enzyme-Linked ImmuoSorbent Assay (ELISA). Samples were pooled per vaccine group and diluted with PBS to achieve the same gD2-specific IgG antibody titer in the Ad35.HSV.gD2 and CJ2-gD2 serum pools (‘High dose’). Two consecutive 3-fold dilutions were generated as well (‘Mid dose’, ‘Low dose’). Three doses were generated per group to cover a dose range that would resemble gD2 IgG ELISA titers induced during an active immunization.

Naïve female BALB/c mice (10 mice/group) were injected i.p three days and two days before challenge with 0.5 ml/day of High, Mid or Low dose of serum from Ad35.HSV.gD2 or CJ2-gD2 vaccine groups. One group of mice received naïve serum (Charles River Benelux) diluted with PBS at the same serum concentration as the ‘High Dose’ serum. A positive control group was also included, where mice had received two doses of Ad35.HSV.gD2 (10^10^ vp/mouse) 6 and 2 weeks before intravaginal challenge. The levels of transferred antibodies were determined on serum collected one day before challenge to evaluate transfer efficiency. Mice were then challenged intravaginally on day 0 with 200LD50 (3.2 x10^4^ pfu/mouse) of WT HSV-2 G strain virus and monitored for 21 days as described above. One animal in the Ad35.HSV.gD2 mid serum dose group, 1 animal in the CJ2-gD2 high serum dose group and 1 animal in the naïve serum group could not be evaluated as they did not survive the challenge procedure. The cause of death is unknown, since the procedure went according to schedule, but the animals did not wake up. Technical issues were reported for the plaque assay for 9 animals in the serum transfer study, generating no data for the correlation analysis depicted in Fig 6.

### Virus neutralization assay (VNA)

Virus neutralizing antibody titers (VNT) in mouse serum were determined against either HSV-2 or HSV-1 [24]. In brief, BHK-ICP6-LacZ cells (ELVIS cells expressing the *E*. *coli* lacZ gene under an HSV-1 ICP6 promoter) were seeded at a confluency of 3,5 x10^5^ cells/ml in 96-well black and white cell-culture plates (Perkin Elmer) one day prior to infection. On the day of infection, serially diluted sera and controls were mixed with 1x10^4^ pfu/ml of HSV-2 G strain virus or HSV-1 KOS virus and incubated for 1 hour at 37°C in the presence of 1% Baby Rabbit complement (Bio-Rad, ‎Hercules, CA‎, USA). Subsequently, serum-virus mixes were transferred to the BHK-ICP6-LacZ cell monolayers and incubated at 37°C, 5% CO2 overnight. When infected with either HSV-1 or HSV-2, BHK-ICP6-LacZ cells start to encode and accumulate β-galactosidase. Finally, Gal-Screen substrate (Thermo Fisher Scientific, Waltham, MA, USA) was added and luminescence was measured by Synergy Neo (BioTek, Winooski, VT, USA). VNA titers were calculated as the antibody concentration that caused a 50% (IC50) reduction in luminescence for HSV-2 and HSV-1 respectively, and titers were expressed as Log2IC50.

### Enzyme linked immunosorbent assay (ELISA)

HSV-2 gD2 specific IgG immune responses were measured by ELISA. Maxisorp 96-well plates (Nunc, Thermo Fisher Scientific) were coated with soluble gD2 protein (produced in HEK293 cells and purified at U-Protein Express, Utrecht, The Netherlands) at a concentration of 0.5 μg/ml and incubated overnight at 4°C. The following day plates were washed with PBS (Invitrogen, Waltham, MA, USA) containing 0.05% Tween-20 (Promega, Madison, WI, USA) and subsequently blocked with PBS containing 2% dried skimmed milk (Becton Dickinson, Franklin Lakes, NJ, USA) for 1 hour at RT. After washing, individual serum samples and the standard (Abcam6507, Abcam, Cambridge, UK) were serially diluted and added in duplicate. Plates were incubated for 1 hour (h) at room temperature (RT) and washed. IgG antibodies were detected by 1h incubation with a Horseradish peroxidase (HRP)-labeled goat anti-Mouse IgG conjugate (1:2000) (SeraCare, Milford, MA) at RT. After washing, the reaction was developed with O-phenylenediamine dihydrochloride (OPD) substrate (Thermo Fisher Scientific) for 10 minutes and the reaction was stopped by 1M H_2_SO_4_ addition. The optical density (OD) was measured at 492 nm. Titers were expressed as the log10 of ug/ml (in ELISA Units, EU/ml) calculated by a weighted average, using the squared slope of the standard curve at the location of each quantification as weight. Negative samples were set at the Lower Limit of Quantification (LLoQ). gD1 ELISA was performed by an identical procedure, using soluble gD1 protein (produced in HEK293 cells and purified at U-Protein Express).

HSV-2 specific gB2 ELISA titers were measured in mouse serum using and Endpoint ELISA. White plates (Greiner Bio-one, Kremsmünster, Austria) were coated at a concentration of 0.25 μg/ml and incubated overnight at 4°C with soluble gB2 protein (produced in HEK cells and purified at U-Protein Express, Utrecht, The Netherlands). After washing, individual serum samples were serially diluted and added in duplicate. Plates were incubated for 1 hour at RT and washed. Antibodies were detected by 1 hour incubation with a Horseradish peroxidase (HRP) labeled goat-anti-Mouse IgG conjugate (1:2000) (SeraCare, Milford, MA) at RT. Lumiglow Chemiluminescent Substrate A and Substrate B was added (SeraCare) and the plates were incubated for 30 min at RT in the dark. Luminescence was measured by Synergy Neo (BioTek) and endpoint titers were calculated by linear interpolation and expressed as Log10 titers.

HSV-2 specific IgG1 and IgG2a antibody titers were measured according to the gB2 ELISA protocol, using gD2 as antigen and HRP-labeled anti-mouse IgG1 and IgG2a (Southern Biotech, Birmingham, AL, USA) as secondary antibodies, both diluted at 1:1000.

### Enzyme-linked immunospot assay (ELISpot)

HSV-2 specific IFN-γ responses were assessed in individual mouse spleens. Splenocytes from mice immunized with Ad35.HSV.gD2 were aseptically collected and processed in R10 medium: RPMI1640 (Invitrogen) supplemented with 10% FBS, 100 units/mL Penicillin/100 μg/mL Streptomycin (Gibco), Minimum Essential Medium (MEM) with 0.01 mM non-essential amino acids (Invitrogen) and 13 μM 2-mercaptoethanol. Splenocytes from mice immunized with CJ2-gD2 were aseptically collected and processed in R0 medium (R10 medium without FBS). Mouse IFN-γ ELISpot kit (MabTech AB, Nacka Strand, Sweden) was used according to the manufacturer’s instructions. Splenocytes were seeded at 2.5x10^5^ cells/well density in duplicate and stimulated with a gD2 peptide pool (JPT, Berlin, Germany) consisting of 15-mer peptides with an 11-amino-acid-overlap, spanning the whole sequence of the surface glycoprotein gD2 of HSV-2. Splenocytes from mice immunized with CJ2-gD2 were stimulated with three pools consisting of 15-mer peptides with an 11-amino-acid-overlap covering the glycoprotein gB2 protein (3 pools: Pool 1:1–93 aa, Pool 2: 94-189aa, Pool 3:196–224 aa), ICP27 (13-mer: AFLTGADRSVRLA) and a peptide from the tegument protein VP11/12 (9-mer: HGPSLYRTF). Negative medium (R10), and positive, PMA (2ng/ml) (phorbol 12-myristate 13-acetate)-ionomycin (2μg/ml), control wells per mouse were also included. After 18±1 h incubation at 37°C, 10% CO2, splenocytes were washed with PBS with 0.05% Tween. Plates were incubated for 2 h with a biotinylated detection mAb R4-6A2 (1:1000) (Mabtech) in PBS with 1% FBS. Plates were washed and incubated for 1 hour at RT with streptavidin-ALP (MabTech) (1:2000) in PBS with 1% FBS. Next, final washing was done before development of specific spot formation with BCIP/NBT substrate (MabTech) between 3 to -6 minutes in the dark. Development was stopped by discarding the development solution and thoroughly washing the plates with dH2O. Spots were quantified with an AELVIS ELISpot reader (AELVIS GmbH). Spot forming Unit (SFU) per 10^6^ cells were calculated and background levels were calculated as the 95% percentile of the SFU observed in non-stimulated splenocytes.

### Statistical analysis

Statistical comparison across dose between the regimens with one or two immunizations of Ad35.HSV.gD2 and between the two highest doses of Ad35.HSV.gD2 and CJ2-gD2 was performed for most readouts by analysis-of-variance (ANOVA) with 2-fold Bonferroni adjustment for multiple comparisons. For HSV-2 titers in vaginal swabs, Wilcoxon pairwise comparisons with 6-fold Bonferroni adjustments were used. Correlation analyses were performed using the Spearman rank correlation method. Statistical analyses were performed using SAS version 9.4 (SAS Institute, Inc., Cary, NC, USA).

## Results

### Immunization with Ad35.HSV.gD2 induces humoral and cellular immune responses in BALB/c mice

Immunogenicity of Ad35.HSV.gD2 was studied in BALB/c mice and an HSV-2 recombinant viral vaccine, CJ2-gD2, was used as a positive control. CJ2-gD2 is an ICP0 mutant-based, non-replicating, dominant-negative recombinant HSV-2. CJ2-gD2 expresses the gD2 antigen as efficiently as the wild type HSV-2 virus and has been shown to induce strong humoral immune responses characterized by virus neutralizing antibodies and protection against HSV-2 infection in mice and guinea pigs [23]. In addition, it has shown superior protective efficacy compared with the MPL/alum adjuvanted gD2 subunit vaccine [25]. As a viral vector, CJ-gD2 contains all relevant antigens that may contribute to protection and as a comparator for immunogenicity and efficacy studies, it can be expected to be superior compared to Ad35.HSV.gD2. It provides the opportunity for improvement on the adenoviral vaccine concept, with more favorable properties, to be clinically developed.

**Fig 1:**
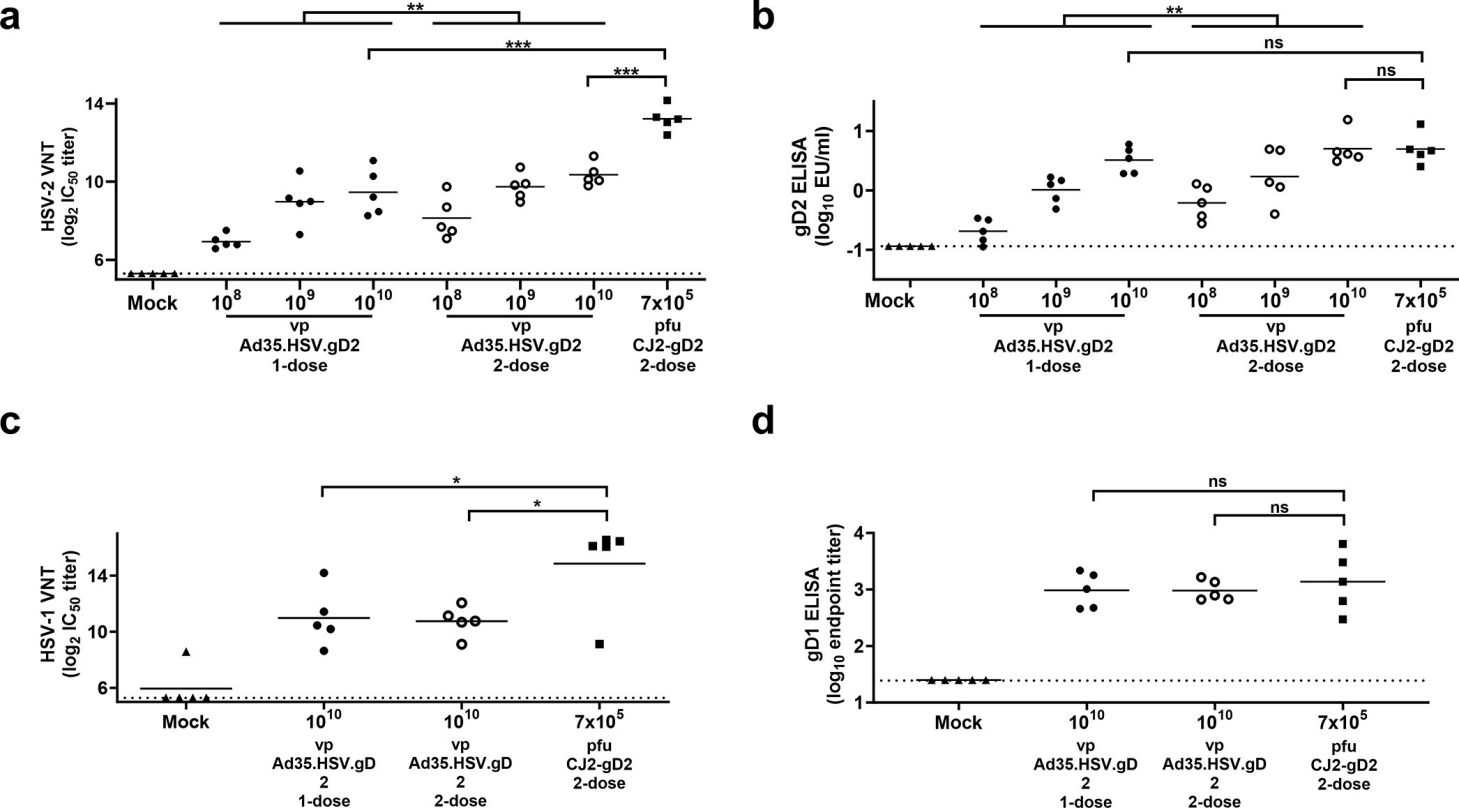
Immunization with Ad35.HSV.gD2 induces HSV-2 specific humoral immune responses in BALB/c mice. Female BALB/c mice were immunized intramuscularly once (T = 0d) or twice (T = 0d and T = 28d) with 10^8^ to 10^10^ vp/mouse Ad35.HSV.gD2 (N = 5/group), twice with 7x10^5^ pfu/mouse CJ2-gD2 (N = 5) (T = 0d and T = 28d) or mock immunized with formulation buffer (N = 5) and sacrificed 6 weeks after the first immunization. Serum samples were collected (T = 42d) and analyzed for (a) HSV-2 Virus Neutralizing antibody titers (shown as log_2_ IC_50_) and (b) gD2 IgG ELISA titers (shown as log10 EU/ml). (c) Cross-reactive neutralizing antibody titers against HSV-1 KOS virus (shown as log_2_ IC_50_) and (d) gD1 IgG ELISA titers (shown as log_10_ endpoint titers) were only measured in sera from mice receiving 2-dose regimens. Dotted lines indicate the lower limit of quantification (LLoQ, gD2 ELISA) or limit of detection (LOD, other assays) and horizontal lines indicate the mean value per group. (a-b) (Across dose) statistical comparison and (c-d) highest dose comparison was performed by ANOVA with Bonferroni correction. Results of statistical analysis are depicted by asterisks: *p<0.05, ** p<0.01, *** p<0.001; ns: not significant.

Mice were immunized intramuscularly with 10^10^, 10^9^, 10^8^ viral particles (vp) of Ad35.HSV.gD2 in either a one-dose or a two-dose regimen or with CJ2-gD2 (7x10^5^ pfu/mouse) in a two-dose regimen as control. A negative control group received two mock immunizations with formulation buffer. Six weeks after the first immunization, humoral immune responses were measured in sera of individual mice. Mice immunized with Ad35.HSV.gD2 showed a dose-dependent antibody response. Significantly higher virus neutralizing antibody titers (VNT) (Fig 1A) and gD2 ELISA titers (Fig 1B) were measured for the two-dose regimen compared to the one dose only (Fig 1A). CJ2-gD2 induced significantly higher VNT in comparison with Ad35.HSV.gD2 (Fig 1A). The presence of additional HSV-2 antigens in the CJ2-gD2 vaccine might explain the overall difference in VNT between these two vaccines platforms (S1 Fig). No significant difference in gD2 ELISA titers was observed between CJ2-gD2 and 10^10^ vp/mouse Ad35.HSV.gD2.

Furthermore, cross-reactive gD1 IgG ELISA titers and HSV-1 VNT were elicited by both Ad35.HSV.gD2 (only the 10^10^ vp dose tested) and CJ2-gD2. No significant difference was observed between the one-dose and two-dose regimens of Ad35.HSV.gD2, although CJ2-gD2 induced significantly higher VNT against HSV-1 virus compared with Ad35.HSV.gD2 (Fig 1C). Comparable gD1 IgG ELISA titers were elicited by Ad35.HSV.gD2 and CJ2-gD2 (Fig 1D).

**Fig 2:**
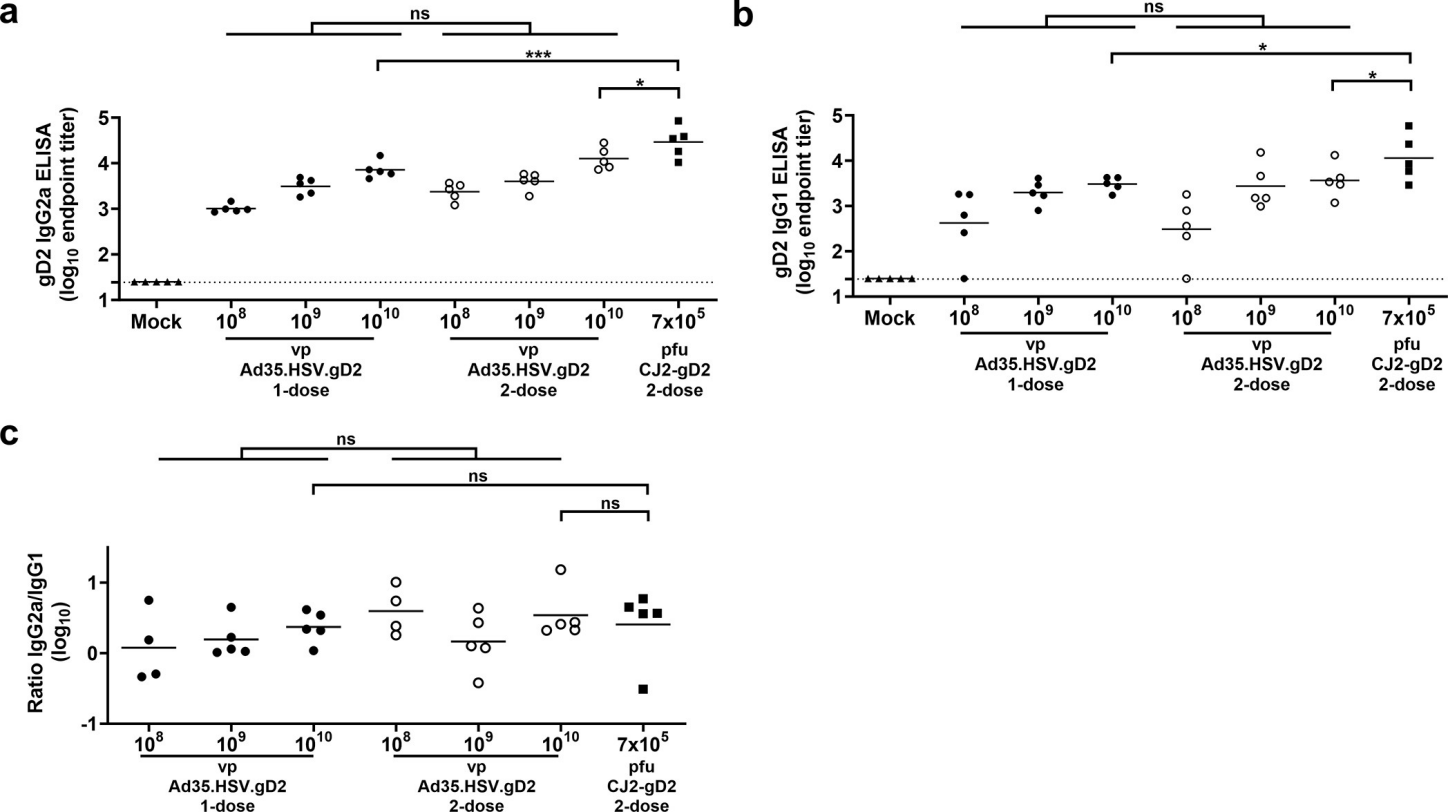
Immunization with Ad35.HSV.gD2 induces gD2 IgG1 and IgG2a subclass ELISA titers. Female BALB/c mice were immunized as described in Fig 1. (a) IgG2a and (b) IgG1 subclass antibodies against the gD2 protein were assessed in serum by ELISA (T = 42d) (shown as log_10_ endpoints titers). (c) Log_10_ IgG2a/IgG1 ELISA titer ratio is shown. Dotted lines indicate the limit of detection (LOD) and horizontal lines indicate the mean response per group. Across dose level comparison between 1- and 2-dose regimens was performed by ANOVA and comparison between highest vaccine dose levels were performed by ANOVA with Bonferroni correction. Results of statistical analysis are depicted by asterisks: *p<0.05, *** p<0.001; ns: not significant.

Both IgG2a (Fig 2A) and IgG1 (Fig 2B) antibodies against the gD2 protein were induced by Ad35.HSV.gD2, and differences between one- and two-dose regimens did not reach statistical significance. Although CJ2-gD2 induced significantly higher antibody levels, no significant difference in IgG2a/IgG1 ELISA titer ratio could be identified between vaccine platforms (Fig 2C), suggesting induction of a comparable T helper 1 (Th1)/T helper 2 (Th2) ratio.

**Fig 3:**
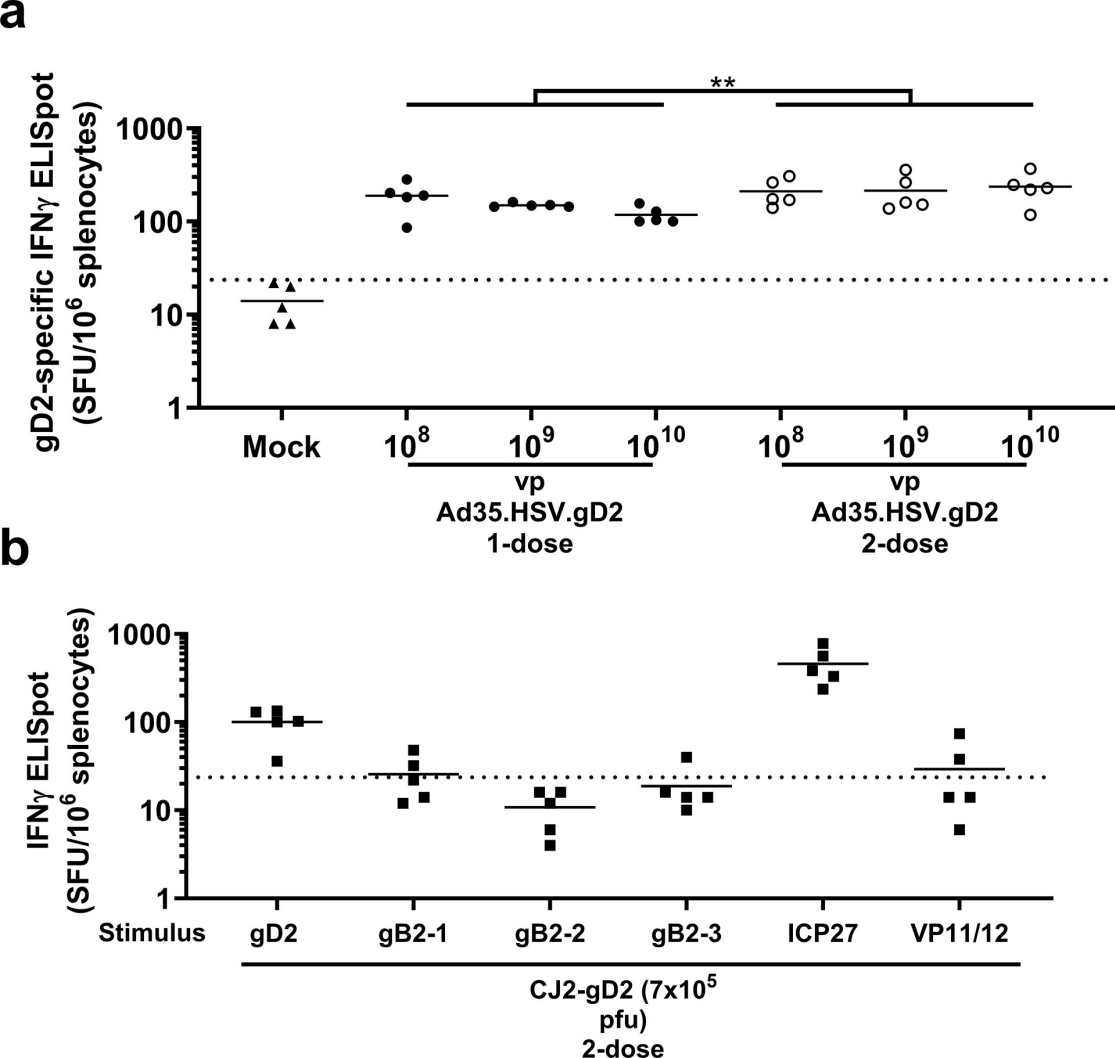
Prime and prime boost immunization with Ad35.HSV.gD2 induces HSV-2 specific IFNγ responses. Female BALB/c mice were immunized as described in Fig 1. Cellular immune responses were assessed by IFN-γ ELISpot on splenocytes harvested from BALB/c mice (T = 42d). (a) Ad35.HSV.gD2 splenocytes were stimulated with a gD2 peptide pool overnight in R10 medium while (b) CJ2-gD2 splenocytes were stimulated in R0 medium with a gD2 peptide pool, gB2 peptide pools (gB-1, gB-2 and gB-3), ICP27 (13-mer: AFLTGADRSVRLA) and VP11/12 (9-mer: HGPSLYRTF). Results are depicted as spot forming units (SFU)/10^6^ splenocytes. Dotted lines indicate the background of the assay (95th percentile of medium stimulated samples) and the horizontal lines depict the geometric mean response per group. Across dose statistical comparison between the one- and 2-dose regimens was performed by ANOVA. Results of statistical analysis are depicted by asterisks: ** p<0.01.

gD2-specific cellular responses were detected in spleen by IFNγ ELISpot after immunization with Ad35.HSV.gD2, although no dose response was observed (Fig 3A). IFNγ responses were also measured for the Ad35 surface antigen hexon as a control in the ELISpot assay (S2 Fig). Since the CJ2-gD2 vaccine contains fetal bovine serum (FBS), which causes background responses in the assay, spleen processing and the assay were performed in medium without FBS, and the results are shown in a separate graph. CJ2-gD2 showed cellular responses against gD2 (to a similar level compared to Ad35.HSV.gD2) and additional antigens expressed by CJ2-gD2: ICP27 and (at a lower level) gB2 and VP11/12 (Fig 3B), suggesting that CJ2-gD2 induces cellular responses against a broad range of HSV antigens (other antigens were not tested).

### Immunization with Ad35.HSV.gD2 induces complete protection against death and reduced viral shedding after HSV-2 intravaginal challenge

**Fig 4:**
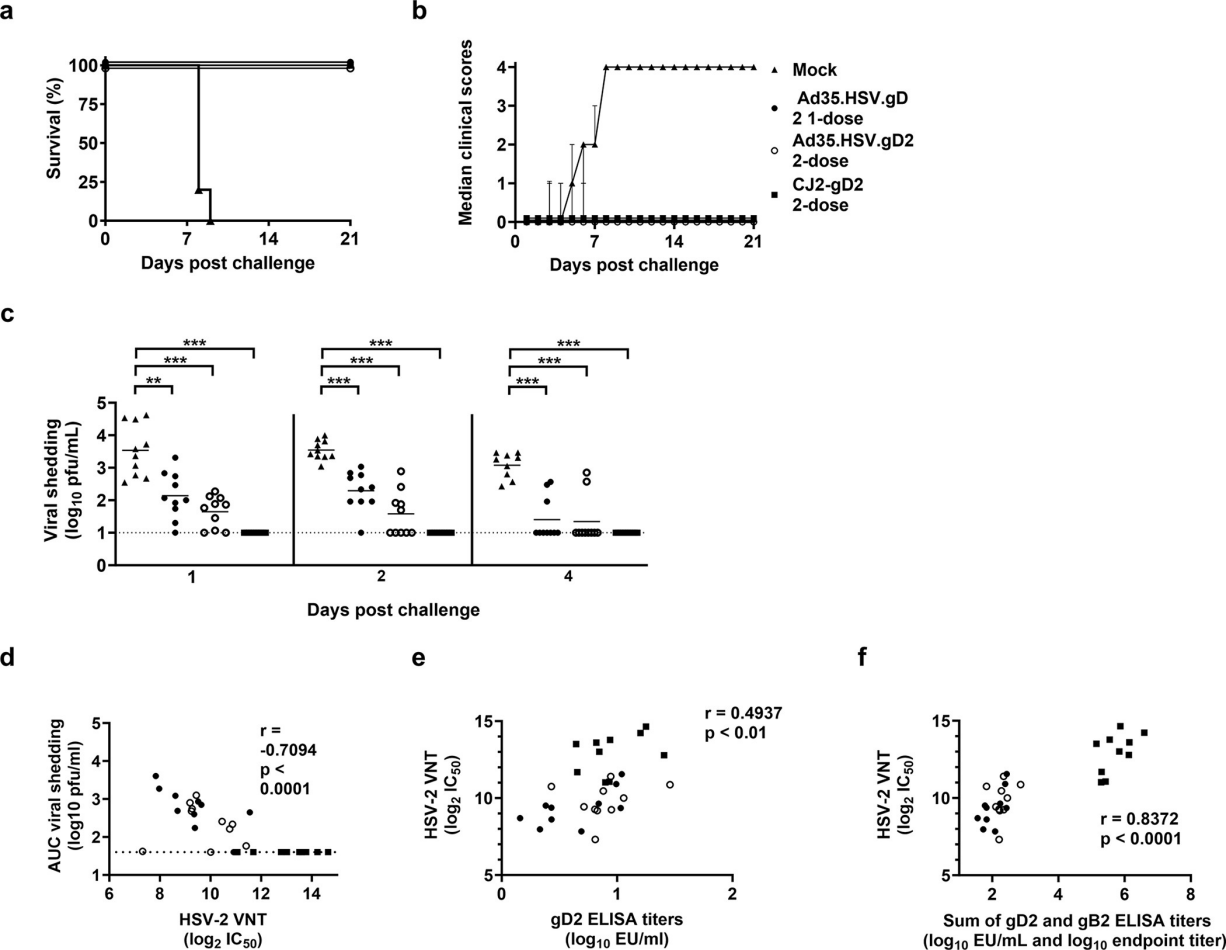
Prime and prime-boost immunization with Ad35.HSV.gD2 induces complete protection and reduced viral shedding in BALB/c mice after HSV-2 intravaginal challenge. Female BALB/c mice were immunized intramuscularly once at T = 0d or twice (T = 0d and T = 28d) with 10^10^ vp/mouse Ad35.HSV.gD2 (N = 10/group), twice with 7x10^5^ pfu/mouse CJ2-gD2 (N = 10) or mock immunized with formulation buffer (N = 10). Mice were intravaginally challenged 6 weeks after the first immunization (T = 42d) with 200LD50 (3.2x10^4^ pfu/mouse) WT HSV-2 G strain and monitored daily for (a) survival and (b) clinical scores over 21 days after infection. (c) Viral shedding was assessed by plaque assay on vaginal swabs collected on days 1, 2 and 4 after challenge (viral titers expressed as log_10_ plaque forming units (pfu)/mL). HSV-2 VNT (titer expressed as log_2_ IC_50_), gD2 ELISA and gB2 ELISA (titer expressed as log_10_ EU/mL) were performed on sera collected 1 day before challenge and correlation analyses were performed between (d) HSV-2 VNT vs viral shedding (Area Under the Curve, AUC) per individual animal on Day 1, 2, 4 (Day 1–4), (e) HSV-2 VNT vs gD2 ELISA titers and (f) HSV-2 VNT vs the sum of gD2 and gB2 ELISA titers. Dotted lines indicate the limit of detection (LOD) and horizontal lines represent mean value per group. Statistical comparisons of viral shedding between immunized groups (c) were performed by a pairwise Wilcoxon test with a 6-fold Bonferroni adjustment. Results of statistical analysis are depicted by asterisks: **p<0.01, *** p<0.001, ns: not significant. Correlation analyses were performed using the Spearman rank correlation method with the correlation coefficient and two-tailed p value for each analysis depicted in the figure.

To study protective efficacy of Ad35.HSV.gD2, we made use of a lethal intravaginal HSV-2 challenge model in mice where we studied the effect on survival, clinical score, and viral shedding. Mice immunized with Ad35.HSV.gD2 or CJ2-gD2 as control received a 200LD50 dose of HSV-2 G strain inoculated intravaginally 2 weeks after the last immunization. Mock immunized mice showed rapid signs of disease progression with disease onset around 2 days after challenge reaching humane endpoint between 7 and 8 days after challenge. Immunized mice showed 100% survival after challenge (Fig 4A) with no clinical symptoms throughout the 21 days monitoring period with exception for some single dosed Ad35.HSV.gD2 immunized animals that showed transient genital edema or erythema between days 3 and 6 after challenge (Fig 4B).

Viral shedding was assessed on intravaginal swabs collected on day 1, 2 and 4 after challenge (Fig 4C). Consistent viral shedding was observed in mock immunized mice at all three time points. All Ad35.HSV.gD2 immunized groups showed detectable viral shedding at all time points, although statistically significantly lower compared with viral shedding in mock immunized mice (Fig 4C). The CJ2-gD2 group showed no detectable viral shedding during the observation period. Humoral immune responses were measured in pre-challenge serum samples of the same animals and comparable humoral responses (VNT and gD2 ELISA titers) were observed as in the immunogenicity study presented in Fig 1. There was a statistically significant inverse correlation between viral shedding in immunized mice at day 1, 2 and 4 (Area Under the Curve, AUC) per mouse and VNT (Fig 4D). A positive correlation was observed between VNT and gD2 ELISA titers (r = 0.4937,**p<0.001, Spearman correlation) (Fig 4E) and correlation increased when gD2 and gB2 ELISA titers were combined (r = 0.8372, ***p<0.0001, Spearman correlation) (Fig 4F), suggesting that antibodies against both antigens contributed to virus neutralization.

### Passive serum transfer provides protection against HSV-2 challenge in mice

**Fig 5:**
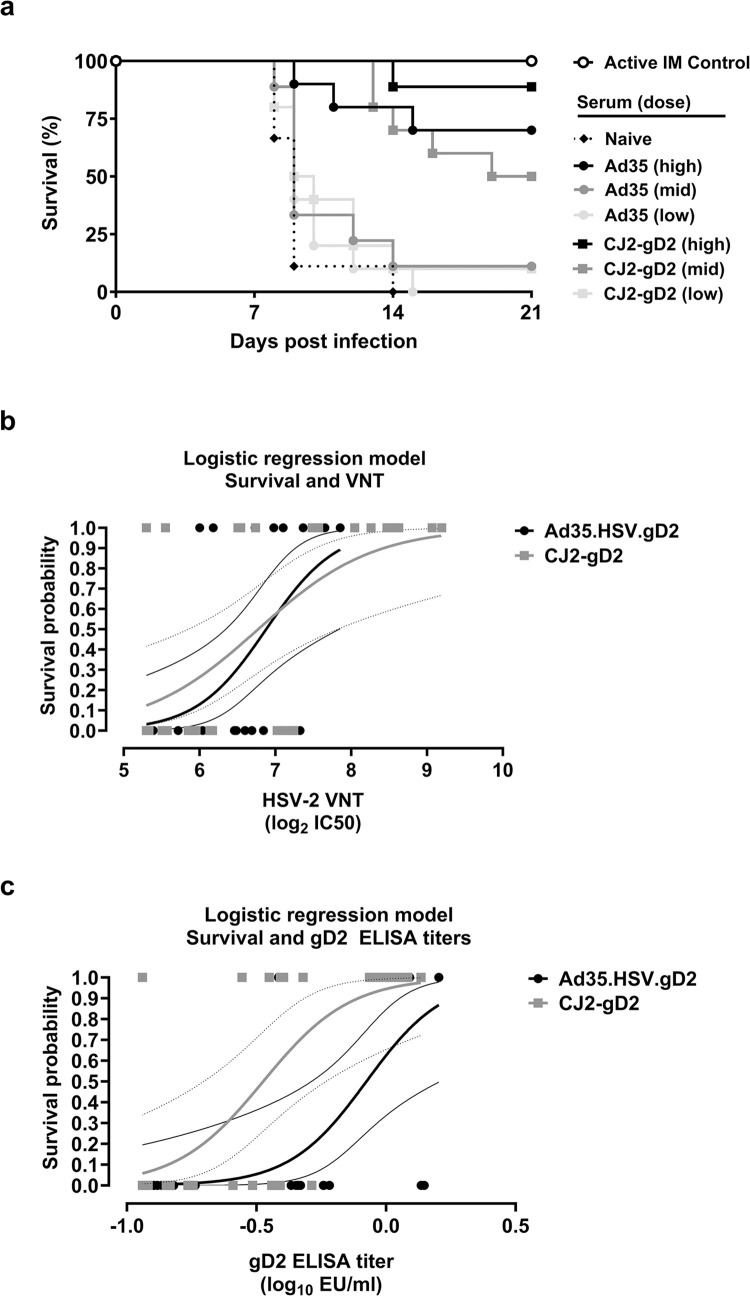
Mice receiving Ad35.HSV.gD2 and CJ2-gD2 immune serum show partial protection and viral shedding after challenge. Mice (N = 10/group) received three dilutions (High, Mid, Low) of serum pools from Ad35.HSV.gD2 or CJ2-gD2 immunized mice 3 and 2 days before intravaginal challenge (0.5 ml serum/mouse/day). Serum pools were diluted to equal gD2 ELISA titers before transfer. Control mice received naïve serum pool or active immunization with Ad35.HSV.gD2 (10^10^ vp/mouse) 42 and 14 days before challenge. Mice were challenged intravaginally with 200LD_50_ HSV-2 G strain on day 0. After challenge mice were monitored daily for (a) survival for 21 days. One day before challenge, serum was collected and HSV-2 VNT and gD2 ELISA titers were measured. Logistic regression analysis was performed using (b) HSV-2 VNT (log_2_ IC_50_) versus survival status (0 = dead at day 21; 1 = alive at day 21) and (c) gD2 ELISA titers (log_10_ EU/mL) versus survival status. Thick black and grey lines depict the regression curves for Ad35.HSV.gD2 and CJ2-gD2, respectively. Thin black lines and thin dotted lines depict the 95% confidence intervals for Ad35.HSV.gD2 and CJ2-gD2, respectively.

To specifically address the protective role of antibodies induced by Ad35.HSV.gD2 against HSV-2, serum from immunized mice was transferred to naïve mice before intravaginal challenge with HSV-2. We transferred serum pools from Ad35.HSV.gD2 and CJ2-gD2 immunized mice at comparable gD2 ELISA titers for comparison of protective efficacy. Serum was diluted before transfer at a gD2 ELISA titer of 0.9 EU/μl and then three-fold dilutions were made before transfer (0.9 EU/μl (High), 0.3 EU/μl (Mid), 0.1 EU/μl (Low)). A negative control group received serum from naïve animals and a positive control group received active immunization with Ad35.HSV.gD2 six and two weeks before challenge. Antibody titers against gD2 were measured in mice 1 day before intravaginal challenge to assess transfer efficiency. Comparable gD2 binding antibody levels were observed between the groups (S3A Fig) and VNT were significantly higher in mice receiving CJ2-gD2 serum in comparison to mice receiving the highest dose of Ad35.HSV.gD2 immune serum (S3B Fig). One day after serum transfer, mice were intravaginally challenged with 200LD50 HSV-2, monitored for survival rate, viral shedding and clinical scores and sacrificed when reaching the humane endpoint. Mice receiving CJ2-gD2 serum showed significantly higher survival (88.8%, 50%, 10% for High, Mid, Low serum dose, respectively) compared with mice receiving Ad35.HSV.gD2 serum (70%, 11.1%, 0% for High, Mid, Low serum dose, respectively) (Fig 5A). The data show that neutralizing antibodies can mediate protection in this mouse model. Logistic regression analysis shows that serum from both Ad35.HSV.gD2 and CJ2-gD2 immunized animals protect at comparable VNT levels (Fig 5B), which did not apply for gD2 ELISA titers (Fig 5C), suggesting that additional vaccine antigens (such as gB2) can contribute to VNT and protection.

**Fig 6:**
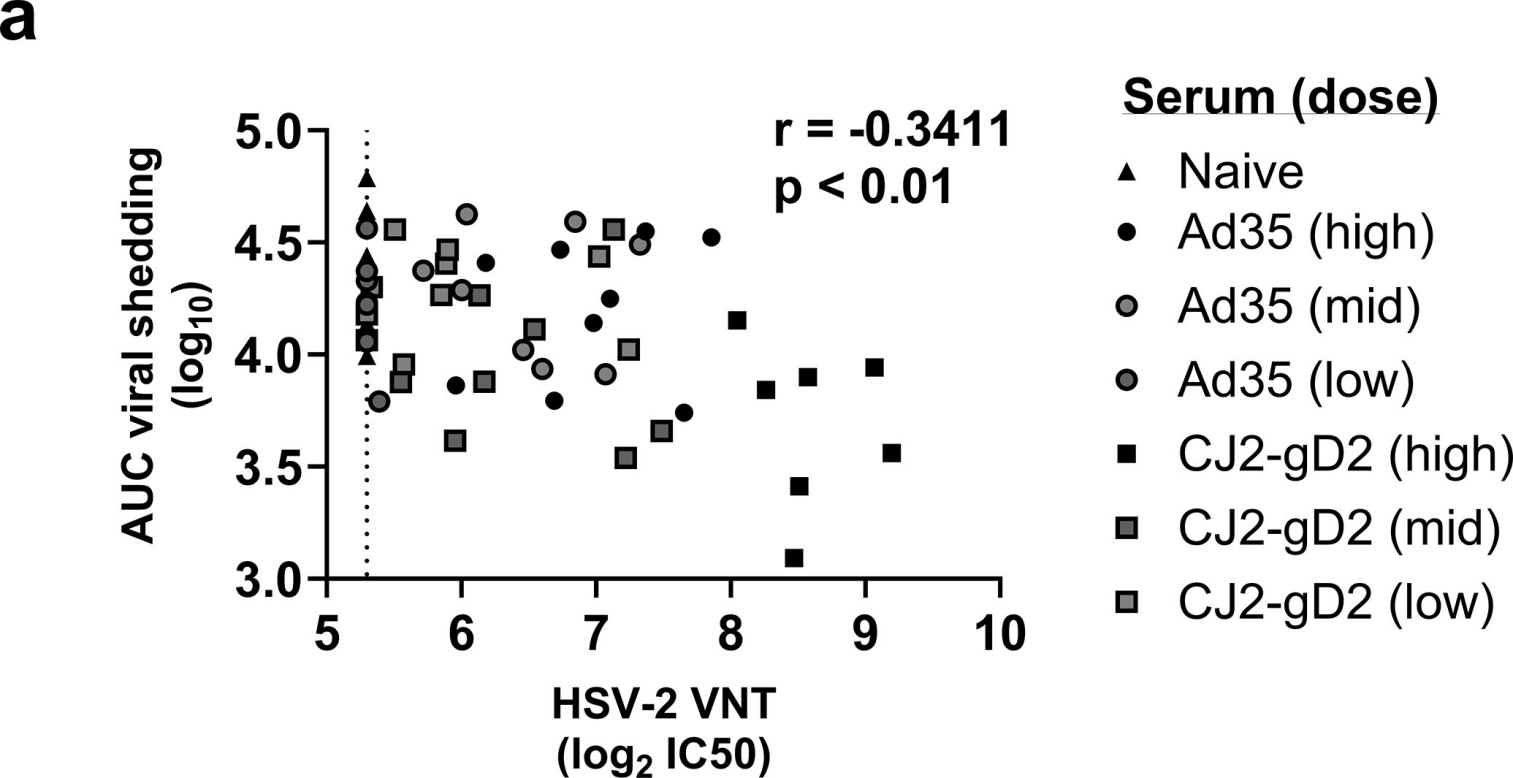
Inverse correlation between HSV-2 VNT and viral shedding in mice receiving immune serum before intravaginal challenge. Mice were passively immunized and challenged intravaginally with HSV-2 as described in Fig 5. Correlation analysis between (a) HSV-2 VNT vs the AUC (Area Under the Curve) viral titer (log_10_) after challenge (Day1-4) are shown. Dotted line depicts the lower limit of quantification (LLOQ). Correlation plot was generated by Spearman rank correlation method resulting in r = -0.3411 (**p<0.01).

All animals receiving serum from vaccinated animals showed viral shedding as measured in intravaginal swabs collected on day 1, 2 and 4 after challenge (S3C Fig) and the titer was overall comparable to the non-treated animals. Control mice receiving active immunization with Ad35.HSV.gD2 showed lower viral shedding, as previously shown (S3C Fig). Significantly lower viral shedding was observed on day 1 and 4 in animals receiving CJ2-gD2 high dose serum compared with mice receiving Ad35.HSV.gD2 high dose serum. Despite the high viral shedding, a significant correlation between viral shedding at day 1, 2 and 4 (AUC) per mouse and VNT was observed (Fig 6).

### Immunization with a combination of Ad35.HSV.gD2 and Ad35.HSV.gB2 shows enhanced protection compared with individual components after HSV-2 intravaginal challenge

**Fig 7:**
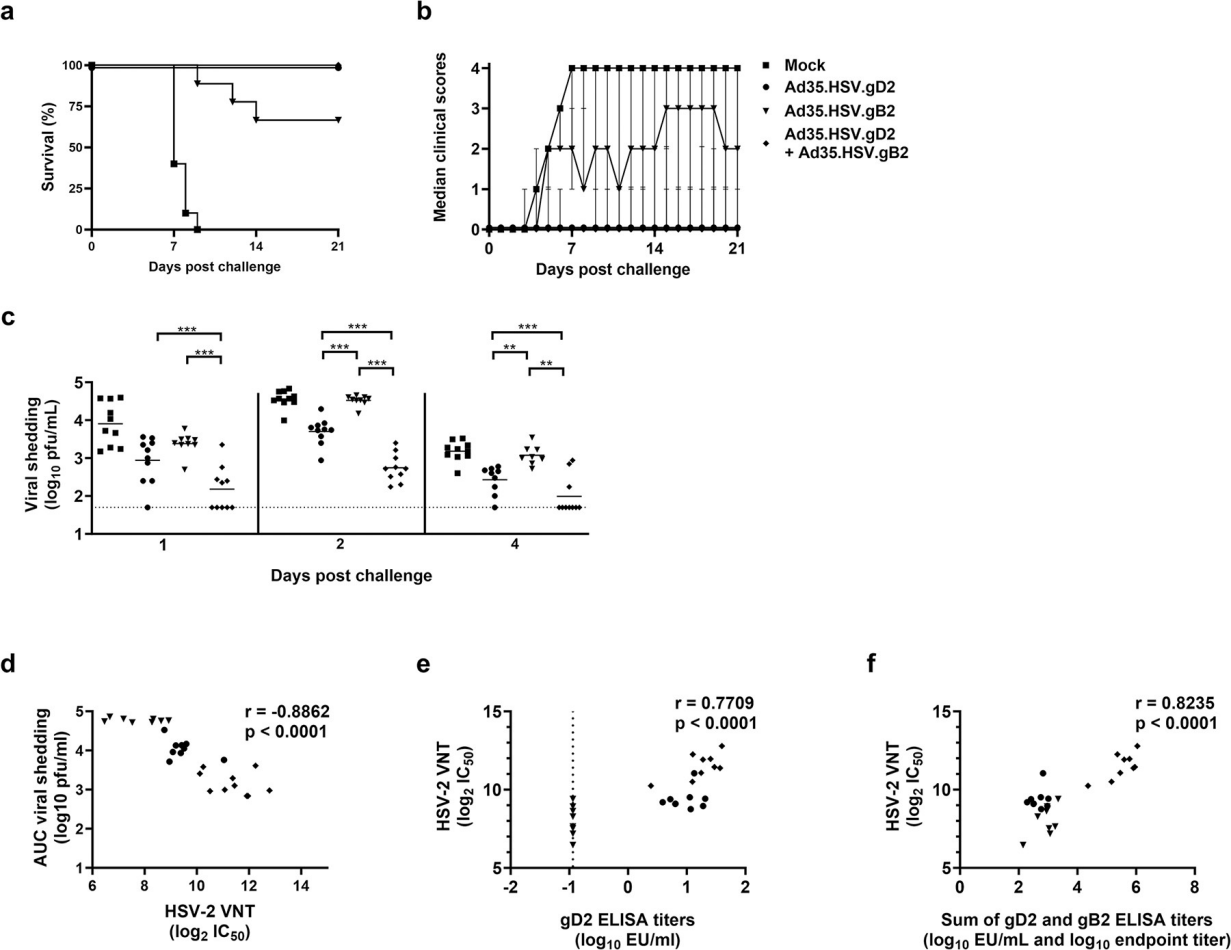
Immunization with a combination of Ad35.HSV.gD2 and Ad35.HSV.gB2 shows enhanced protection compared with individual components after HSV-2 intravaginal challenge. Female BALB/c mice were immunized intramuscularly once at T = 0d with Ad35.HSV.gD2, Ad35.HSV.gB2 (10^10^ vp/mouse, N = 10/group), Ad35.HSV.gD2/ Ad35.HSV.gB2 (10^10^ vp/vector, N = 10/group) or mock immunized with formulation buffer (N = 10). Mice were intravaginally challenged 6 weeks after the first immunization (T = 42d) with 200LD50 (3.2x10^4^ pfu/mouse) WT HSV-2 G strain and monitored daily for (a) survival and (b) clinical scores over 21 days after infection. (c) Viral shedding was assessed by plaque assay on vaginal swabs collected on days 1, 2 and 4 after challenge (viral titers expressed as log_10_ plaque forming units (pfu)/mL). HSV-2 VNT (titer expressed as log_2_ IC_50_), gD2 ELISA and gB2 ELISA (titer expressed as log_10_ EU/mL) were performed on sera collected 1 day before challenge and correlation analyses were performed between (d) HSV-2 VNT vs viral shedding (Area Under the Curve, AUC) per individual animal on Day 1, 2, 4 (Day 1–4), (e) HSV-2 VNT vs gD2 ELISA titers and (f) HSV-2 VNT vs the sum of gD2 and gB2 ELISA titers. Dotted lines indicate the limit of detection (LOD) and horizontal lines represent mean value per group. Statistical comparisons of viral shedding between immunized groups (c) were performed by a pairwise Wilcoxon test with a 6-fold Bonferroni adjustment. Results of statistical analysis are depicted by asterisks: **p<0.01, *** p<0.001, ns: not significant. Correlation analyses were performed using the Spearman rank correlation method with the correlation coefficient and two-tailed p value for each analysis depicted in the figure.

To address the question if additional antigens would enhance protective efficacy of the adenoviral vector vaccine, mice were immunized with a combination of vectors encoding for gD2 (Ad35.HSV.gD2) and gB2 (Ad35.HSV.gB2) and efficacy compared with immunization with each vector alone. Immunized mice were challenged with a 200LD50 dose of HSV-2 G strain inoculated intravaginally 2 weeks after immunization. Mock immunized mice showed rapid signs of disease progression with disease onset around 3 days after challenge reaching humane endpoint between 7 to 9 days after challenge. Mice immunized with Ad35.HSV.gD2 and the combination of two vectors showed 100% survival after challenge (Fig 7A) with no clinical symptoms throughout the 21 days monitoring period with exception for some animals showing genital edema and erythema or moderate genital inflammation (Fig 7B). Mice immunized with Ad35.HSV.gB2 alone showed 67% survival after challenge and the clinical score of surviving animals remained on average between 1 (genital edema and erythema) and 3 (purulent genital lesions or ulcers) during the 21 days of disease monitoring. Consistent viral shedding was observed in mock immunized mice at all time points and viral shedding was detectable in all immunized groups. Viral shedding was significantly lower in animals receiving combination of Ad35.HSV.gD2 and Ad35.HSV.gB2 compared with each vector alone (Fig 7C). There was a statistically significant inverse correlation between viral shedding in immunized mice at day 1, 2 and 4 (AUC) per mouse and VNT (Fig 7D). A positive correlation was observed between VNT and gD2 ELISA titers (r = 0.7709,***p<0.0001, Spearman correlation) (Fig 7E) and between VNT and gD2/gB2 ELISA titers combined (r = 0.8235, ***p<0.0001, Spearman correlation) (Fig 7F). These results demonstrate that combining gD2 and gB2 antigens significantly improves protection compared with individual vectors alone.

## Discussion

There is an unmet medical need to develop a preventive HSV-2 vaccine that can induce robust humoral and T cell mediated protective immune responses. In this study we have characterized the immune responses in mice after immunization with a replication-incompetent Adenoviral vector 35 based HSV-2 vaccine (Ad35.HSV.gD2), with a transgene encoding for gD2. A replication incompetent viral vaccine, CJ2-gD2, was used as a comparator vaccine. Ad35.HSV.gD2 induced robust virus neutralizing antibodies against HSV-2 and HSV-1, with corresponding gD2 (HSV-2) and gD1 (HSV-1) IgG ELISA titers. Antigen-specific IFNγ responses were induced in splenocytes. Immunization resulted in complete survival and reduced viral shedding after intravaginal challenge in a stringent murine HSV-2 infection model. In contrast, no viral shedding was observed after immunization with the CJ2-gD2 vaccine, suggesting that a combination of antigens may improve efficacy of the vaccines. Indeed, combined delivery of Ad35.HSV.gD2 with an adenoviral vector encoding for gB2 (Ad35.HSV.gB2) resulted in a significantly reduced viral shedding compared with Ad35.HSV.gD2 or Ad35.HSV.gB2 alone after intravaginal challenge.

Delivery of a transgene encoding for an immunogen, through replication-incompetent adenoviral vectors, has been demonstrated to be highly potent for induction of humoral and cellular immune responses [22] and, to our knowledge, only limited work has been done on adenoviral vector-based HSV-2 vaccines, with a focus on Ad5-vectored vaccines [26–28]. Ad5 vectors encoding for gD2 and truncated UL25 (containing T cell epitopes) [26] or gB2 [28] have been shown to be immunogenic and protective against HSV challenge in mice. The disadvantage of the Ad5 vector system is the high prevalence of pre-existing immunity in humans, reducing vaccine potency to induce a cellular immune responses [29] and, therefore, not considered suitable for human use. We used Adenoviral vector type 35 (Ad35) due to its low seroprevalence in humans [30]. The Ad35 vector has been shown to elicit robust immune responses in mice and non-human primates [21, 31], also in the presence of Ad5 pre-existing immunity [32].

In the current study, a single immunization with 10^10^ vp Ad35.HSV.gD2 was highly immunogenic while a second immunization only modestly increased the elicited humoral and cellular immune responses. This is possibly due to anti-vector immunity induced by the first immunization that may reduce the potency of the second dose in this rodent model [30, 32]. Anti-vector immunity may be the reason why only 60–70% survival was observed in mice three-times immunized with the Ad5 vector vaccine [26].

In humans and non-human primates, impact of anti-vector immunity, either naturally occurring or elicited by primary vaccination with the same vector-based vaccine was minimal possibly because human adenoviral vectors are better adapted to escaping immune clearance in a (more) native host [33]. Adeno-based COVID-19 vaccines have been associated with a very rare but serious side effect of Vaccine Induced Thrombosis with Thrombocytopenia (VITT). The underlying pathogenic mechanism is unknown but it seems multifactorial [34, 35]. To date, no cases of VITT have been identified in Janssen clinical studies in the RSV vaccine program nor in other (non-COVID-19) Ad26 vectored vaccine programs including Ebola, HIV, HPV, and Zika vaccine development programs or during the Ebola vaccination campaigns. To investigate the contribution of HSV-2 specific antibodies in mediating protection, we transferred immune serum to naïve mice before HSV-2 challenge confirming the important role of antibodies for protection in our challenge model, similar to what has been observed by others in a similar model in BALB/c mice [36]. Logistic regression analysis demonstrated that VNT was an important correlate of protection, where sera from Ad35.HSV.gD2 and CJ2-gD2 immunized mice provided protection at a comparable level. The regression analysis also demonstrated that antibodies against other HSV-2 target antigens, such as gB2, could contribute to protection induced by the replication-defective CJ2-gD2 vaccine, since CJ2-gD2 elicited protection at lower gD2 ELISA titers compared to Ad35.HSV.gD2. Immunization with a combination of Ad35.HSV.gD2 with Ad35.HSV.gB2 resulted in significantly reduced viral shedding in mice after intravaginal challenge with HSV-2, supporting the hypothesis that these antigens can be combined for improved protective efficacy.

These data suggest a contrast with some previously published data where a gB2 subunit vaccine has been shown to provide limited protection against HSV-2 [37] or has not shown additional protective efficacy when combined with gD2 [37–39]. Similarly, additive value of the gB2 protective efficacy component in combination with gD2 has been shown to be low in guinea pigs [37]. This may be related to the presentation of the gB2 vaccine component in these studies, since several studies have demonstrated that monoclonal antibodies against gB2 and gD2 protect mice against intravaginal challenge with HSV-2 [40–45]. Furthermore, a vaccine based on a single-cycle gD2 deleted HSV-2 has shown good protection in mice correlating with non-gD2 specific antibody-dependent cell-mediated cytotoxicity [46]. More research is needed to understand the relative contribution of HSV-2 antigens in the protection from HSV challenge in pre-clinical models and how this may translate to efficacy in humans.

It is clear, though, that neutralizing antibodies appear to play an important role in protection in preclinical animal models. However, these animal models have failed to predict the outcome of clinical trials before [10, 47], suggesting that other antibody characteristics may be required for protective efficacy in humans. More recent studies have shown that antibodies engaging Fcγ receptors on immune cells, such as Natural Killer cells or phagocytes, could play an important role in the protection against HSV-2 infection [46, 48]. Therefore, it has been proposed that antibodies induced by the subunit vaccines used in the clinical trials potentially lacked the necessary effector functions [15]. In contrast, adenoviral vector-based vaccines have been shown to elicit antibodies with Fc-mediated effector functions [49]. We here demonstrate that immunization with both Ad35.HSV.gD2 and CJ2-gD2 induced IgG2a, an IgG subclass that is known to effectively engage Fcγ receptors for the induction of Antibody Dependent Cellular Cytotoxicity (ADCC).

Passive transfer of serum clearly demonstrates the protective effect of antibodies against HSV-2 challenge and a strong correlation was observed between the level of VNT and protection. The Ad35.HSV.gD2 actively immunized group, included in the serum transfer experiment (S3 Fig), showed 100% survival and lower viral shedding compared with the groups passively receiving immune serum. The higher level of protection in actively immunized animals could be contributed by the higher gD2 antibody levels, but the role of HSV-2 specific T cells (induced by Ad35.HSV.gD2 and CJ2-gD2) for the additional level of protection can also not be excluded. Others have shown by T cell depletion experiments (also using CD4 and CD8 T cell knockout mice) that cellular immune responses can play an important role in clearance of vaginal HSV-2 infection in mice [50–52]. The broader cellular immune response (including ICP27 and VP11/12, known to be strong T cell inducers [53]) and humoral immune response induced by CJ2-gD2 likely contributes to the higher level of protective efficacy as observed in the current study.

In conclusion, we have demonstrated that vector mediated delivery of a transgene encoding the HSV-2 gD2 antigen can be an effective immunization strategy against genital herpes. However, as results with the HSV-2 recombinant viral vaccine demonstrated in this study, it is likely that a combination of antigens will be required for most optimal prophylactic efficacy.

## Supporting information

S1 Fig gB2 ELISA titers are induced by CJ2-gD2. (PDF)

S2 Fig IFNγ ELISpot responses against Ad35 surface antigen Hexon were measured as a control in the assay. (PDF)

S3 Fig Antibody levels in mice passively immunized with Ad35.HSV.gD2 and CJ2-gD2 immune serum. (PDF)

## References

[1] Looker KJ, Magaret AS, Turner KM, Vickerman P, Gottlieb SL, Newman LM. Global estimates of prevalent and incident herpes simplex virus type 2 infections in 2012. PLoS One. 2015;10(1):e114989. doi: 10.1371/journal.pone.0114989 25608026 PMC4301914

[2] Roberts C. Genital herpes in young adults: changing sexual behaviours, epidemiology and management. Herpes. 2005;12(1):10–4. 16026639

[3] Tronstein E, Johnston C, Huang ML, Selke S, Magaret A, Warren T, et al. Genital shedding of herpes simplex virus among symptomatic and asymptomatic persons with HSV-2 infection. JAMA. 2011;305(14):1441–9. doi: 10.1001/jama.2011.420 21486977 PMC3144252

[4] Looker KJ, Magaret AS, May MT, Turner KME, Vickerman P, Newman LM, et al. First estimates of the global and regional incidence of neonatal herpes infection. Lancet Glob Health. 2017;5(3):e300–e9. doi: 10.1016/S2214-109X(16)30362-X 28153513 PMC5837040

[5] Corey L, Wald A, Celum CL, Quinn TC. The effects of herpes simplex virus-2 on HIV-1 acquisition and transmission: a review of two overlapping epidemics. J Acquir Immune Defic Syndr. 2004;35(5):435–45. doi: 10.1097/00126334-200404150-00001 15021308

[6] Whitley RJ. Herpes simplex virus infection. Semin Pediatr Infect Dis. 2002;13(1):6–11. doi: 10.1053/spid.2002.29752 12118847

[7] Awasthi S, Hook LM, Shaw CE, Friedman HM. A trivalent subunit antigen glycoprotein vaccine as immunotherapy for genital herpes in the guinea pig genital infection model. Hum Vaccin Immunother. 2017;13(12):2785–93. doi: 10.1080/21645515.2017.1323604 28481687 PMC5718817

[8] Halford WP, Geltz J, Gershburg E. Pan-HSV-2 IgG antibody in vaccinated mice and guinea pigs correlates with protection against herpes simplex virus 2. PLoS One. 2013;8(6):e65523. doi: 10.1371/journal.pone.0065523 23755244 PMC3675040

[9] Skoberne M, Cardin R, Lee A, Kazimirova A, Zielinski V, Garvie D, et al. An adjuvanted herpes simplex virus 2 subunit vaccine elicits a T cell response in mice and is an effective therapeutic vaccine in Guinea pigs. J Virol. 2013;87(7):3930–42. doi: 10.1128/JVI.02745-12 23365421 PMC3624190

[10] Belshe RB, Leone PA, Bernstein DI, Wald A, Levin MJ, Stapleton JT, et al. Efficacy results of a trial of a herpes simplex vaccine. N Engl J Med. 2012;366(1):34–43. doi: 10.1056/NEJMoa1103151 22216840 PMC3287348

[11] Stanberry LR, Spruance SL, Cunningham AL, Bernstein DI, Mindel A, Sacks S, et al. Glycoprotein-D-adjuvant vaccine to prevent genital herpes. N Engl J Med. 2002;347(21):1652–61. doi: 10.1056/NEJMoa011915 12444179

[12] Belshe RB, Heineman TC, Bernstein DI, Bellamy AR, Ewell M, van der Most R, et al. Correlate of immune protection against HSV-1 genital disease in vaccinated women. J Infect Dis. 2014;209(6):828–36. doi: 10.1093/infdis/jit651 24285844 PMC3935479

[13] Koelle DM, Posavad CM, Barnum GR, Johnson ML, Frank JM, Corey L. Clearance of HSV-2 from recurrent genital lesions correlates with infiltration of HSV-specific cytotoxic T lymphocytes. J Clin Invest. 1998;101(7):1500–8. doi: 10.1172/JCI1758 9525993 PMC508728

[14] Nelson MH, Bird MD, Chu CF, Johnson AJ, Friedrich BM, Allman WR, et al. Rapid clearance of herpes simplex virus type 2 by CD8+ T cells requires high level expression of effector T cell functions. J Reprod Immunol. 2011;89(1):10–7. doi: 10.1016/j.jri.2011.01.013 21444117 PMC3081923

[15] Mahant AM, Guerguis S, Blevins TP, Cheshenko N, Gao W, Anastos K, et al. Failure of Herpes Simplex Virus Glycoprotein D Antibodies to Elicit Antibody-Dependent Cell-Mediated Cytotoxicity: Implications for Future Vaccines. J Infect Dis. 2022;226(9):1489–98. doi: 10.1093/infdis/jiac284 35834278 PMC10205893

[16] Krishnan R, Stuart PM. Developments in Vaccination for Herpes Simplex Virus. Front Microbiol. 2021;12:798927. doi: 10.3389/fmicb.2021.798927 34950127 PMC8691362

[17] Lee CS, Bishop ES, Zhang R, Yu X, Farina EM, Yan S, et al. Adenovirus-Mediated Gene Delivery: Potential Applications for Gene and Cell-Based Therapies in the New Era of Personalized Medicine. Genes Dis. 2017;4(2):43–63. doi: 10.1016/j.gendis.2017.04.001 28944281 PMC5609467

[18] Tatsis N, Ertl HC. Adenoviruses as vaccine vectors. Mol Ther. 2004;10(4):616–29. doi: 10.1016/j.ymthe.2004.07.013 15451446 PMC7106330

[19] Mercado NB, Zahn R, Wegmann F, Loos C, Chandrashekar A, Yu J, et al. Single-shot Ad26 vaccine protects against SARS-CoV-2 in rhesus macaques. Nature. 2020;586(7830):583–8. doi: 10.1038/s41586-020-2607-z 32731257 PMC7581548

[20] Vogels R, Zuijdgeest D, van Meerendonk M, Companjen A, Gillissen G, Sijtsma J, et al. High-level expression from two independent expression cassettes in replication-incompetent adenovirus type 35 vector. J Gen Virol. 2007;88(Pt 11):2915–24. doi: 10.1099/vir.0.83119-0 17947512

[21] Widjojoatmodjo MN, Bogaert L, Meek B, Zahn R, Vellinga J, Custers J, et al. Recombinant low-seroprevalent adenoviral vectors Ad26 and Ad35 expressing the respiratory syncytial virus (RSV) fusion protein induce protective immunity against RSV infection in cotton rats. Vaccine. 2015;33(41):5406–14. doi: 10.1016/j.vaccine.2015.08.056 26319741

[22] Zahn R, Gillisen G, Roos A, Koning M, van der Helm E, Spek D, et al. Ad35 and ad26 vaccine vectors induce potent and cross-reactive antibody and T-cell responses to multiple filovirus species. PLoS One. 2012;7(12):e44115. doi: 10.1371/journal.pone.0044115 23236343 PMC3516506

[23] Akhrameyeva NV, Zhang P, Sugiyama N, Behar SM, Yao F. Development of a glycoprotein D-expressing dominant-negative and replication-defective herpes simplex virus 2 (HSV-2) recombinant viral vaccine against HSV-2 infection in mice. J Virol. 2011;85(10):5036–47. doi: 10.1128/JVI.02548-10 21389121 PMC3126160

[24] Blevins TP, Mitchell MC, Korom M, Wang H, Yu Y, Morrison LA, et al. Higher Throughput Quantification of Neutralizing Antibody to Herpes Simplex Viruses. PLoS One. 2015;10(12):e0144738. 10.1371/journal.pone.0144738 PMC4682838 26658766

[25] Zhang P, Xie L, Balliet JW, Casimiro DR, Yao F. A herpes simplex virus 2 (HSV-2) glycoprotein D-expressing nonreplicating dominant-negative HSV-2 virus vaccine is superior to a gD2 subunit vaccine against HSV-2 genital infection in guinea pigs. PLoS One. 2014;9(6):e101373. doi: 10.1371/journal.pone.0101373 24979708 PMC4076306

[26] Liu W, Zhou Y, Wang Z, Zhang Z, Wang Q, Su W, et al. Evaluation of recombinant adenovirus vaccines based on glycoprotein D and truncated UL25 against herpes simplex virus type 2 in mice. Microbiol Immunol. 2017;61(5):176–84. doi: 10.1111/1348-0421.12482 28378925

[27] Wan M, Yang X, Sun J, Ding X, Chen Z, Su W, et al. An Adenovirus-Based Recombinant Herpes Simplex Virus 2 (HSV-2) Therapeutic Vaccine Is Highly Protective against Acute and Recurrent HSV-2 Disease in a Guinea Pig Model. Viruses. 2023; 15(1):219. doi: 10.3390/v15010219 36680259 PMC9861952

[28] McDermott MR, Graham FL, Hanke T, Johnson DC. Protection of mice against lethal challenge with herpes simplex virus by vaccination with an adenovirus vector expressing HSV glycoprotein B. Virology. 1989;169(1):244–7. doi: 10.1016/0042-6822(89)90064-0 2538036

[29] Zak DE, Andersen-Nissen E, Peterson ER, Sato A, Hamilton MK, Borgerding J, et al. Merck Ad5/HIV induces broad innate immune activation that predicts CD8^+^ T-cell responses but is attenuated by preexisting Ad5 immunity. PNAS. 2012; 109(50):E3503–12. 23151505 10.1073/pnas.1208972109 PMC3528489

[30] Abbink P, Lemckert AA, Ewald BA, Lynch DM, Denholtz M, Smits S, et al. Comparative seroprevalence and immunogenicity of six rare serotype recombinant adenovirus vaccine vectors from subgroups B and D. J Virol. 2007;81(9):4654–63. doi: 10.1128/JVI.02696-06 17329340 PMC1900173

[31] Salisch NC, Izquierdo Gil A, Czapska-Casey DN, Vorthoren L, Serroyen J, Tolboom J, et al. Adenovectors encoding RSV-F protein induce durable and mucosal immunity in macaques after two intramuscular administrations. NPJ Vaccines. 2019;4:54. doi: 10.1038/s41541-019-0150-4 31885877 PMC6925274

[32] Barouch DH, Pau MG, Custers JH, Koudstaal W, Kostense S, Havenga MJ, et al. Immunogenicity of recombinant adenovirus serotype 35 vaccine in the presence of pre-existing anti-Ad5 immunity. J Immunol. 2004;172(10):6290–7. doi: 10.4049/jimmunol.172.10.6290 15128818

[33] Khan S, Salisch NC, Gil AI, Boedhoe S, Boer KF, Serroyen J, et al. Sequential use of Ad26-based vaccine regimens in NHP to induce immunity against different disease targets. NPJ Vaccines. 2022;7(1):146. doi: 10.1038/s41541-022-00567-w 36379957 PMC9664441

[34] Struyf F, Hardt K, Van Rampelbergh R, Shukarev G, Inamdar A, Ruiz-Guinazu J, et al. Thrombosis with thrombocytopenia syndrome: A database review of clinical trial and post-marketing experience with Ad26.COV2.S. Vaccine. 2023;41(37):5351–9. doi: 10.1016/j.vaccine.2023.07.013 37517912

[35] Greinacher A, Schonborn L, Siegerist F, Steil L, Palankar R, Handtke S, et al. Pathogenesis of vaccine-induced immune thrombotic thrombocytopenia (VITT). Semin Hematol. 2022;59(2):97–107. doi: 10.1053/j.seminhematol.2022.02.004 35512907 PMC8863951

[36] Parr EL, Parr MB. Immunoglobulin G is the main protective antibody in mouse vaginal secretions after vaginal immunization with attenuated herpes simplex virus type 2. J Virol. 1997;71(11):8109–15. doi: 10.1128/JVI.71.11.8109-8115.1997 9343160 PMC192266

[37] Bernstein DI, Earwood JD, Bravo FJ, Cohen GH, Eisenberg RJ, Clark JR, et al. Effects of herpes simplex virus type 2 glycoprotein vaccines and CLDC adjuvant on genital herpes infection in the guinea pig. Vaccine. 2011;29(11):2071–8. doi: 10.1016/j.vaccine.2011.01.005 21238569 PMC3082315

[38] Lee HH, Cha SC, Jang DJ, Lee JK, Choo DW, Kim YS, et al. Immunization with combined HSV-2 glycoproteins B2: D2 gene DNAs: protection against lethal intravaginal challenges in mice. Virus Genes. 2002;25(2):179–88. doi: 10.1023/a:1020113902834 12416680

[39] McClements WL, Armstrong ME, Keys RD, Liu MA. Immunization with DNA vaccines encoding glycoprotein D or glycoprotein B, alone or in combination, induces protective immunity in animal models of herpes simplex virus-2 disease. Proc Natl Acad Sci U S A. 1996;93(21):11414–20. doi: 10.1073/pnas.93.21.11414 8876149 PMC38071

[40] Balachandran N, Bacchetti S, Rawls WE. Protection against lethal challenge of BALB/c mice by passive transfer of monoclonal antibodies to five glycoproteins of herpes simplex virus type 2. Infect Immun. 1982;37(3):1132–7. doi: 10.1128/iai.37.3.1132-1137.1982 6290390 PMC347658

[41] Daumer MP, Schneider B, Giesen DM, Aziz S, Kaiser R, Kupfer B, et al. Characterisation of the epitope for a herpes simplex virus glycoprotein B-specific monoclonal antibody with high protective capacity. Med Microbiol Immunol. 2011;200(2):85–97. doi: 10.1007/s00430-010-0174-x 20931340

[42] Dix RD, Pereira L, Baringer JR. Use of monoclonal antibody directed against herpes simplex virus glycoproteins to protect mice against acute virus-induced neurological disease. Infect Immun. 1981;34(1):192–9. doi: 10.1128/iai.34.1.192-199.1981 6271681 PMC350842

[43] Eis-Hubinger AM, Schmidt DS, Schneweis KE. Anti-glycoprotein B monoclonal antibody protects T cell-depleted mice against herpes simplex virus infection by inhibition of virus replication at the inoculated mucous membranes. J Gen Virol. 1993;74 (Pt 3):379–85. doi: 10.1099/0022-1317-74-3-379 8383173

[44] Hook LM, Cairns TM, Awasthi S, Brooks BD, Ditto NT, Eisenberg RJ, et al. Vaccine-induced antibodies to herpes simplex virus glycoprotein D epitopes involved in virus entry and cell-to-cell spread correlate with protection against genital disease in guinea pigs. PLoS Pathog. 2018;14(5):e1007095. doi: 10.1371/journal.ppat.1007095 29791513 PMC5988323

[45] Kuraoka M, Aschner CB, Windsor IW, Mahant AM, Garforth SJ, Kong SL, et al. A non-neutralizing glycoprotein B monoclonal antibody protects against herpes simplex virus disease in mice. J Clin Invest. 2023;133(3). doi: 10.1172/JCI161968 36454639 PMC9888390

[46] Petro CD, Weinrick B, Khajoueinejad N, Burn C, Sellers R, Jacobs WR Jr., et al. HSV-2 DeltagD elicits FcgammaR-effector antibodies that protect against clinical isolates. JCI Insight. 2016;1(12). 10.1172/jci.insight.88529 PMC4985247 27536733

[47] Corey L, Langenberg AG, Ashley R, Sekulovich RE, Izu AE, Douglas JM Jr., et al. Recombinant glycoprotein vaccine for the prevention of genital HSV-2 infection: two randomized controlled trials. Chiron HSV Vaccine Study Group. JAMA. 1999;282(4):331–40. doi: 10.1001/jama.282.4.331 10432030

[48] Chu CF, Meador MG, Young CG, Strasser JE, Bourne N, Milligan GN. Antibody-mediated protection against genital herpes simplex virus type 2 disease in mice by Fc gamma receptor-dependent and -independent mechanisms. J Reprod Immunol. 2008;78(1):58–67. doi: 10.1016/j.jri.2007.08.004 17950908 PMC2441821

[49] Alter G, Yu WH, Chandrashekar A, Borducchi EN, Ghneim K, Sharma A, et al. Passive Transfer of Vaccine-Elicited Antibodies Protects against SIV in Rhesus Macaques. Cell. 2020;183(1):185–96 e14. doi: 10.1016/j.cell.2020.08.033 33007262 PMC7534693

[50] Dudley KL, Bourne N, Milligan GN. Immune protection against HSV-2 in B-cell-deficient mice. Virology. 2000;270(2):454–63. 10793004 10.1006/viro.2000.0298

[51] Milligan GN, Bernstein DI. Interferon-gamma enhances resolution of herpes simplex virus type 2 infection of the murine genital tract. Virology. 1997;229(1):259–68. doi: 10.1006/viro.1997.8441 9123869

[52] Milligan GN, Bernstein DI, Bourne N. T lymphocytes are required for protection of the vaginal mucosae and sensory ganglia of immune mice against reinfection with herpes simplex virus type 2. J Immunol. 1998;160(12):6093–100. 9637526

[53] Muller WJ, Dong L, Vilalta A, Byrd B, Wilhelm KM, McClurkan CL, et al. Herpes simplex virus type 2 tegument proteins contain subdominant T-cell epitopes detectable in BALB/c mice after DNA immunization and infection. J Gen Virol. 2009;90(Pt 5):1153–63. doi: 10.1099/vir.0.008771-0 19264627 PMC2675279

