# Supplementary material for: A replication-incompetent adenoviral vector encoding for HSV-2 gD2 is immunogenic and protective against HSV-2 intravaginal challenge in mice
Source: PLoS One. 2024 Dec 31;19(12):e0310250. doi: 10.1371/journal.pone.0310250 (PMC11687876; doi:10.1371/journal.pone.0310250)
Supplement: S1 Fig — (PDF) [file pone.0310250.s001.pdf]

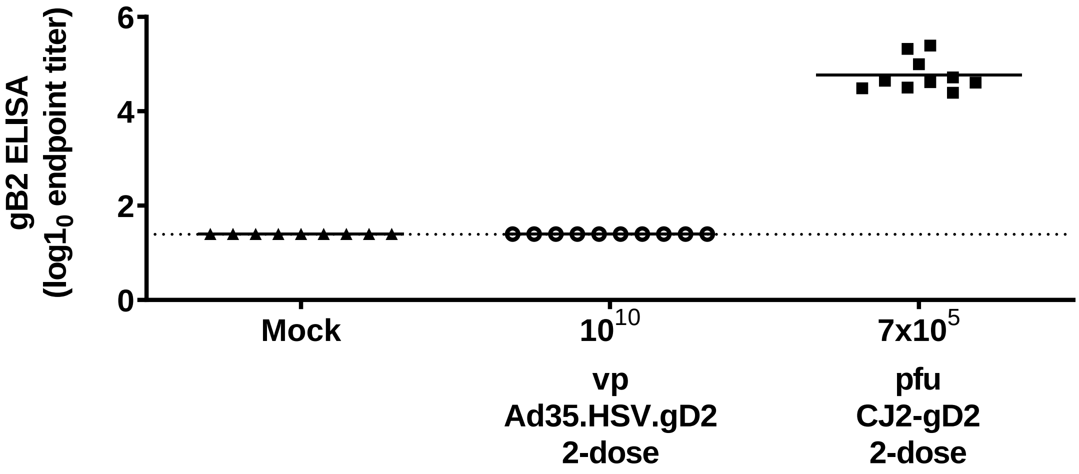

Supplementary Figure 1. gB2 ELISA titers are induced by CJ2-gD2

Female BALB/c mice were immunized intramuscularly twice (T=0d and T=28d) with 10^{10} vp/mouse with Ad35.HSV.gD2 (N=10/group) or twice (T=0d and T=28d) with $7x10^5$ pfu/mouse CJ2-gD2 (N=10) or mock immunized with formulation buffer (N=10) and sacrificed 6 weeks after the first immunization. Serum samples were collected (T=42d) and analyzed for gB2 IgG ELISA titers (shown as log10 endpoint titers). Dotted line indicates the limit of detection (LOD) and horizontal lines indicate the mean value per group.
