# Supplementary material for: A replication-incompetent adenoviral vector encoding for HSV-2 gD2 is immunogenic and protective against HSV-2 intravaginal challenge in mice
Source: PLoS One. 2024 Dec 31;19(12):e0310250. doi: 10.1371/journal.pone.0310250 (PMC11687876; doi:10.1371/journal.pone.0310250)
Supplement: S2 Fig — (PDF) [file pone.0310250.s002.pdf]

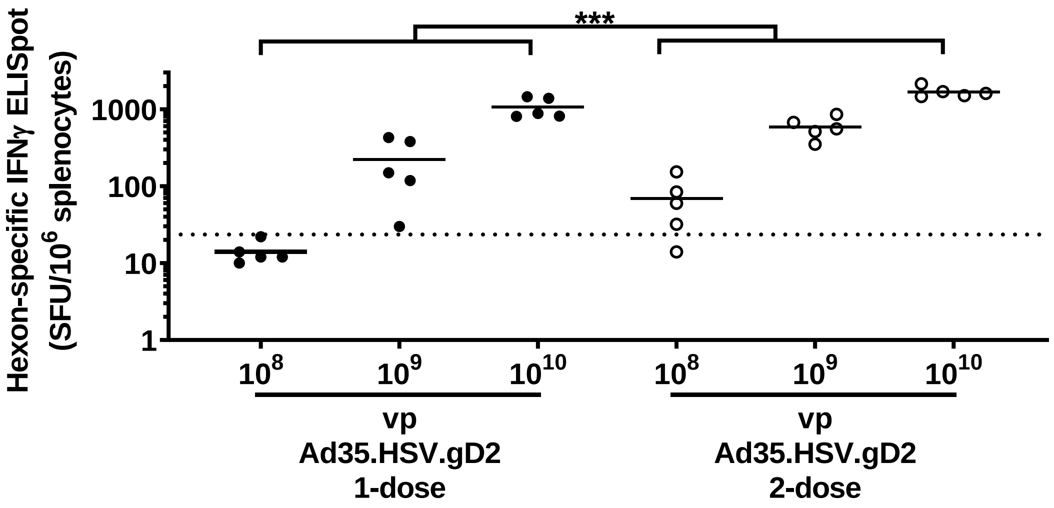

Supplementary Figure 2. IFNγ ELISpot responses against Ad35 surface antigen Hexon were measured as a control in the assay.

Female BALB/c mice were immunized intramuscularly once (T=0d) or twice (T=0d and T=28d) 10^8 to 10^{10} vp/mouse with Ad35.HSV.gD2 (N=5/group) or twice (T=0d and T=28d) with $7x10^5$ pfu/mouse CJ2-gD2 (N=5) or mock immunized with formulation buffer (N=5) and sacrificed 6 weeks after the first immunization. Cellular immune responses were assessed by IFN-γ ELISpot on splenocytes harvested from BALB/c mice (T=42d). Ad35.HSV.gD2 splenocytes were stimulated with an Ad35 immunodominant hexon peptide (9-mer: KYTPSNVTL) overnight in R10 medium, as a control for the ELISpot assay presented in Figure 3. Dose responses curves were observed for mice immunized with Ad35.HSV.gD2. Results are depicted as spot forming units (SFU)/10^6 splenocytes. Dotted lines indicate the background of the assay (95th percentile of medium stimulated samples) and the horizontal lines depict the geometric mean response per group. Across

dose statistical comparison between the 1-dose vs 2-dose regimen was performed by ANOVA. Results of statistical analysis are depicted by asterisks: *$p<0.05$, *** $p<0.001$; results $p>0.05$ (not significant) are not shown.
