# Supplementary material for: A replication-incompetent adenoviral vector encoding for HSV-2 gD2 is immunogenic and protective against HSV-2 intravaginal challenge in mice
Source: PLoS One. 2024 Dec 31;19(12):e0310250. doi: 10.1371/journal.pone.0310250 (PMC11687876; doi:10.1371/journal.pone.0310250)
Supplement: S3 Fig — (PDF) [file pone.0310250.s003.pdf]

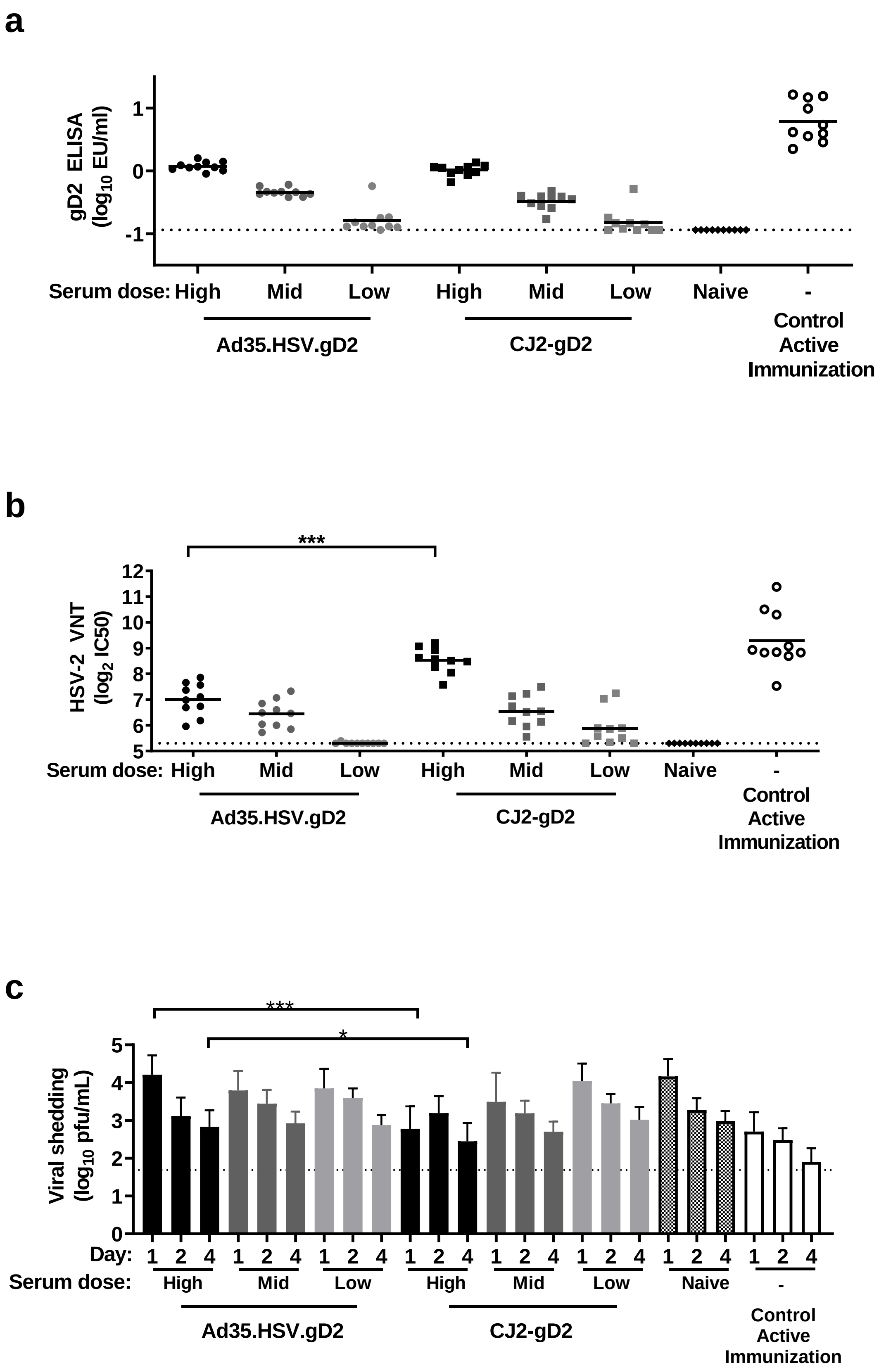
a
gD2 ELISA
(log_{10} EU/ml)
1
0
-1
Serum dose: High
Mid
Low
High
Mid
Low
Naive
-
Ad35.HSV.gD2
CJ2-gD2
Control
Active
Immunization
b
***
HSV-2 VNT
(log_2 IC50)
12
11
10
9
8
7
6
5
Serum dose: High
Mid
Low
High
Mid
Low
Naive
-
Ad35.HSV.gD2
CJ2-gD2
Control
Active
Immunization
c
***
*
Viral shedding
(log_{10} pfu/mL)
5
4
3
2
1
0
Day: 1 2 4 1 2 4 1 2 4 1 2 4 1 2 4 1 2 4 1 2 4 1 2 4
Serum dose: High
Mid
Low
High
Mid
Low
Naive
-
Ad35.HSV.gD2
CJ2-gD2
Control
Active
Immunization

Supplementary figure 3. Antibody levels in mice passively immunized with Ad35.HSV.gD2 and CJ2-gD2 immune serum.

Naïve Balb/C mice (N=10/group) received three dilutions (High, Mid, Low) of serum pools from Ad35.HSV.gD2 or CJ2-gD2 immunized mice, respectively, 3 and 2 days before intravaginal challenge (0.5 ml serum/mouse/day). Serum pools were diluted to equal gD2 ELISA titers before serum transfer. Negative control mice received naïve serum pool. Positive control mice were actively immunized with Ad35.HSV.gD2 (10^{10} vp/mouse) 42 and 14 days before challenge. Mice were challenged intravaginally with 200LD50 HSV-2 G strain on day 0. Serum was collected at day -1 before challenge and (**a**) gD2 ELISA titers and (**b**) virus neutralization titers (VNT) were measured. (**c**) Viral shedding (shown as log10 pfu/ml) was assessed by plaque assay on vaginal swabs collected on days 1, 2 and 4 after challenge. Dotted lines indicate the lower limit of quantification (LLoQ) or limit of detection (LOD) and horizontal lines indicate the mean value per group. (**a-c**) (Across dose) statistical comparison and (**a-c**) highest dose comparison was performed by ANOVA with Bonferroni correction. Results of statistical analysis are depicted by asterisks: *p<0.05, *** p<0.001; results p>0.05 (not significant) are not shown.
